# IMPACT OF BARIATRIC SURGERY IN ANXIETY AND ORAL CONDITION OF OBESE INDIVIDUALS: A COHORT PROSPECTIVE STUDY

**DOI:** 10.1590/0102-672020210002e1615

**Published:** 2021-12-17

**Authors:** Adriana Maria Fuzer Grael TINÓS, Gerson Aparecido FORATORI-JUNIOR, Wagner MARCENES, Felipe Borges CAMARGO, Francisco Carlos GROPPO, Silvia Helena de Carvalho SALES-PERES

**Affiliations:** 1 University of São Paulo, Faculty of Dentistry of Bauru, Department of Pediatric Dentistry, Orthodontics and Public Health, Bauru, SP, Brazil; 2 King's College London, Public Health, London, WE, England; 3 Community Health Centre, Public Health System, São Paulo, SP, Brazil; 4 University of Campinas, Department of Physiological Sciences, Piracicaba, SP, Brazil

**Keywords:** Anxiety Disorders, Bariatric surgery, Dental caries, Obesity, morbid, Periodontal Diseases, Transtornos de Ansiedade, Cirurgia bariátrica, Cárie Dentária, Obesidade mórbida, Doenças Periodontais

## Abstract

**Background:**

Obesity and bariatric surgery may be related with mental and oral disorders.

**Aim:**

To evaluate the impact of bariatric surgery on anxiety, initial dental caries lesion and gingival bleeding in obese patients.

**Methods:**

Eighty-nine patients were divided in two groups: Control Group (CG) - obese patients and Experimental Group (EG) - patients submitted to bariatric surgery. EG was analyzed before and 12 months after bariatric surgery; for the CG, was respected an interval of 12 months between the evaluations. International Caries Detection and Assessment System, Gingival Bleeding Index and Trace-State Anxiety Inventory were used. Medical profile, anthropometrics data, sociodemographic and behavioral variables were considered.

**Results:**

There were no statistically significant differences between groups in evaluation times regarding to initial dental caries lesion and anxiety. However, the number of teeth with initial dental caries lesion (p=0.0033) and gingival bleeding (p<0.0001) increased significantly after bariatric surgery in EG.

**Conclusion:**

These results reinforce the need for multi-professional team follow-up, including dental care, for both obese and bariatric patients.

## RESUMO

**Racional:**

A obesidade e a cirurgia bariátrica podem estar relacionadas a desordens mentais e bucais.

**Objetivo:**

Avaliar o impacto da cirurgia bariátrica na ansiedade, lesões iniciais de cárie dentária e sangramento gengival em pacientes obesos.

**Método:**

Oitenta e nove pacientes foram divididos em dois grupos: Grupo Controle (GC) - obesos e Grupo Experimental (GE) - pacientes submetidos à cirurgia bariátrica. O GE foi analisado antes e 12 meses após a cirurgia bariátrica; para o GC, foi respeitado um intervalo de 12 meses entre as avaliações. Foram utilizados o Sistema Internacional de Detecção e Avaliação de Cárie, Índice de Sangramento Gengival e Inventário de Ansiedade do Traço-Estado.

**Resultados:**

Não houve diferenças estatisticamente significantes entre os grupos nos dois momentos de avaliação em relação à lesão inicial de cárie dentária e ansiedade. No entanto, o número de dentes com lesão inicial de cárie dentária (p=0,0033) e sangramento gengival (p<0,0001) aumentou significativamente após a cirurgia bariátrica no GE.

**Conclusão:**

É necessário acompanhamento de equipe multiprofissional, incluindo atendimento odontológico, tanto para pacientes obesos quanto para bariátricos.

## INTRODUCTION

Oral health may be associated with both obesity and anxiety. Obesity and most of other oral diseases share some etiological factors^25^, such as high frequency of food intake and unhealthy eating habits. Psychological disorders, including anxiety, may be related to low frequency of visits to the dentist, neglected oral hygiene, use of tobacco and unhealthy diets^13^.

The high prevalence of obesity and the lack of satisfactory results in its conservative treatments have led to an increase in the number of bariatric surgeries in western countries^14, 23, 27^. It is important to consider that individuals submitted to bariatric surgery present lifestyle changes, especially in relation to food consumption. Due to the fact these patients feed more times a day in small portions, they may have an increase in dental plaque formation. Moreover, if they do not perform a good oral hygiene, there may be dental caries and gingival alterations^19, 25^.

Several researchers sought to analyze the influence of bariatric surgery on oral health^11^ ^,^ ^21^. However, there is a lack of studies investigating this association using, as outcomes, dental caries in their early stages and gingival bleeding, which are potentially reversible. Furthermore, there are few studies that investigated, simultaneously, these oral problems, obesity, anxiety, and the influence of bariatric surgery on these disorders, and the interference of these variables in weight loss after the procedure.

 The alternative hypothesis of the present study was that there is improvement in anxiety levels after bariatric surgery. Nevertheless, there is a worsening of oral condition regarding to the initial dental caries lesion and gingival bleeding of obese patients, candidates for bariatric surgery in Brazilian Public Health System.

 Thus, the main objective of this study was to evaluate the impact of bariatric surgery on anxiety, on initial dental caries lesion and on gingival bleeding in obese patients submitted to bariatric surgery in the public health service. Moreover, the secondary objective was to compare these results with the ones of patients who were not submitted to bariatric surgery.

## METHOD

The STROBE guidelines were used to ensure the reporting of this observational longitudinal prospective cohort study^26^. It was developed by the Department of Pediatric Dentistry, Orthodontics and Public Health of the Bauru School of Dentistry of the University of São Paulo and Gastroenterology and Bariatric Surgery Ambulatory of Hospital Amaral Carvalho, located in the Jaú city, SP, Brazil.

### Ethical standards

In respect to the Declaration of Helsinki guidelines, this study was submitted and approved by Ethics Committee on Human Research (n^o^ 06/2016). All subjects signed a written consent form regarding their participation in the study.

### Participants

A team of dentists assisted 100 obese patients, at the Brazilian Public Health System, intending to undergo bariatric surgery, that were divided into two groups: Experimental Group (EG) and Control Group (CG).

A pilot study with 31 patients was performed to calculate the sample. These patients of pilot study did not participate in the present study. For each outcome, a sample was calculated, adopted the highest number. Significance level of 5%, test power of 80% and minimally significant clinical difference of 30% were adopted. There was an increase of 20% in the sample, taking into account the possibility of eventual losses during this longitudinal study.

Inclusion criteria were age between 18 and 60 years, body mass index (BMI) equal or higher than 40 kg/m² or 35 kg/m² with presence of obesity-related comorbidities, history of obesity greater than five years with failure of previous conventional treatments for weight loss, absence of previous bariatric surgery and presence of at least seven dental elements.

### Study design

The sample recruitment was performed during the first dental attendance of the patients at Bauru School of Dentistry. After recruitment, patients were monitored regarding scheduling for surgery. The first 50 patients who had their scheduled surgeries would be distributed to the EG.

In order to standardize the diagnostic criteria that involve oral evaluations and to minimize variations between the same and different examiners, the team of dentists was previously calibrated. The calibration process was conducted by a standard examiner, experienced in epidemiological surveys. Every ten examinations there was a duplicate. Kappa test (>0.75) was used to measure agreement, whose interpretation was based on the criteria proposed by Landis & Koch^15^.

Patients from both groups were evaluated in two moments. The EG was evaluated before bariatric surgery (T0) and one year after the procedure (T1). For the CG, the interval of one year between the evaluations was respected. At T0, the application of the anxiety questionnaire occurred at the moment of recruitment, by a specialist in the area. The sociodemographic and behavioral data, the anthropometric measures [weight (kg) and height (meters)], the use of psychiatric medication, the presence of diabetes and/or hypertension as well as the information on oral conditions of interest were collected through a review of the medical records of the selected patients.

In T1, the evaluation of the oral condition and anxiety was performed by the researcher and by a professional in the area, respectively, at the time of the postoperative medical appointments of one year for EG and follow-up for CG. After the appointments, the weight data were collected by a review of the medical records. The period of data collection was from July/2013 (during recruitment was performed the first evaluation - T0) to June/2015.

### Examinations

The sociodemographic variables considered in this study were age (in years), gender (male and female), color (white, black and brown), schooling (Elementary School complete or incomplete; High School complete or incomplete; Higher Education complete or incomplete), monthly family income regarding minimum wage defined by Brazilian government (=1; from 1 to 2; from 2 to 3; from 3 to 4; from 4 to 5; >5) and number of residents in the house (up to two, three, four, five and six or higher). Behavioral variables refer to smoking, alcoholism, use of psychiatric medication, that were measured of dichotomous form (yes/no) and number of daily dental brushings (once, twice, three times and four times or more). It was also considered the presence of diabetes and hypertension.

The International Caries Detection and Assessment System (ICDAS II) was used to evaluate initial dental caries lesion (IDCL), which allows the identification of initial lesions without cavitation. When the enamel is evaluated, the difference is clinically detectable through drying^12^. For the analysis, ICDAS II mean was also considered (sum of the scores divided by the number of teeth examined, therefore, the higher result means the worse condition) and the presence of IDCL.

For gingival condition evaluation, Gingival Bleeding Index (GBI) was used, which refers to the presence or absence of bleeding after gingival sulcus probing. To perform gingival probing, it was used periodontal probe (North Carolina-type manual periodontal probe - HU-FRIEDY). Positive GBI was considered when bleeding occurred within 10 s after probing. For the calculation of this index, the dental element was considered, and the number of positive findings was expressed as a percentage, in relation to the number of teeth present. All teeth present in the mouth were examined, except for third molars.

To measure the anxiety of the sample, in T0 and T1, the State-Trait Anxiety Inventory (STAI) was applied. It corresponds to a self-assessment questionnaire composed of two distinct scales elaborated to measure two concepts of anxiety: the state and the trait^10^. The choice of STAI was due to the fact that it is the most complete and used instrument in the studies aiming to evaluate anxiety.

The initial patients' weight and height were collected by a previously trained nurse. In T1, the weight was measured during the medical appointment. These data were used to calculate the BMI, obtained by dividing the weight (kg) by the height (m) raised squared.

### Statistical analysis

Descriptive analysis of the data was performed by calculating means (±Standard Deviation) and medians (first and third quartiles) for quantitative variables. For categorical variables, absolute and relative frequencies were obtained.The data were initially submitted to the distribution analysis by the Kolmogorov-Smirnov test and the variance homoscedasticity test (Levene's test). For the sociodemographic and behavioral data analysis, Fisher's exact test and Chi-square test was used, and the Mann-Whitney test was applied for the height. Weight and BMI, as well as anxiety (STAI state and trace), were analyzed by the two-way ANOVA test. The Kruskal-Wallis test was used to evaluate the numerical variables related to the oral condition. Chi-square and Fisher's exact tests were used for the categorical variables [presence of IDCL and gingival bleeding (GB)]. Linear regression analysis was conducted to analyze the dependence of BMI and weight loss with the variables of this study. Finally, to determine if bariatric surgery was considered a risk factor for ICL and GB, the Relative Risk (RR) was calculated, considering Confidence Interval (95% CI) and p value. Moreover, the differences between the Incidence Rates (IR) of both outcomes regarding the two groups (p value) were considered.

## RESULTS

The final sample of study consisted of 89 participants (EG: 46 and CG: 43). In the initial period (T0), 50 subjects were evaluated in each group, but during the follow-up, in the CG 07 individuals had to be excluded from the sample. Among them, three underwent bariatric surgery in other services, two became total edentulous and two gave up participating in the study. In EG, one became total edentulous and three gave up participating.

Regarding to the demographic and behavioral profile of both groups, in T0, there were no statistically significant differences between groups in any of the variables evaluated. Most of the participants were aged 31 to 40 years (38.2%). Moreover, they had completed second degree of schooling (36%) and a monthly family income of two to three minimum wages (31.5%). The majority of the sample was composed of women (88.4% and 84.8%) in both groups.

Concerning to anthropometric data, there were no statistically significant differences (Mann-Whitney, p=0.16) among the height medians (1^st^ and 3^rd^ quartiles) of CG (1.61, 1.57 - 1.66 meters) and EG (1.65, 1.58-1.68 meters). There was also no difference in CG's weight and BMI between the evaluations. However, analysis of the data (ANOVA at two criteria) revealed that there was a significant decrease (p<0.05) concerning weight and BMI in EG after bariatric surgery. Initial weight and BMI values did not differ between the two groups.

Considering the BMI classification, 37.2% of CG remained morbidly obese and 34.9% of them remained superobese, after one year. It is important to highlight there was an improvement in the BMI condition, from T0 to T1, in 16.3% of the participants. Among them 4.7% morbid obesity (grade III) became grade II obese, and yet, 11.6% of the superobese in T0 become morbid obesity in T1.On the other hand, 4.7% of morbid obese in T0 changed to superobese in T1. In EG, only one subject (2.2%) remained superobese. Of the other patients, 10.9% EG's morbid obese and superobese (in T0) turn out to be grade II obese, 41.3% became obese and 34.7% became non-obese. Two patients (4.3%), who initially were classified as grade II obese, left the obesity condition after one year after surgery.

Regarding anxiety, there were no statistically significant differences (ANOVA at two criteria) between groups and evaluation times, nor the STAI-State (p = 0.69) nor the STAI-Trait (p = 0.52).

Regarding the variables related to the oral condition, the CG had the highest ICDAS II median, in T0. However, in T1, the highest median was identified in the EG, both compared to the CG during the same period and concerning the same group, in T0. In the CG, between periods, there was no significant difference regarding the number of healthy teeth. Nevertheless, in T0, CG presented this lower measure when compared to EG (p=0.0079). The final evaluation (T1) between two groups did not show statistically significant differences. The number of teeth with initial dental caries lesion (IDCL) increased significantly (p=0.0013) after bariatric surgery, as well as the percentage of gingival bleeding (p=0.0029) and the number of teeth with gingival bleeding (p=0.0096, Table 1).

**TABLE 1:** Experimental (EG) and control (CG) groups’ oral conditions, in T0 and T1

Variable	Evaluation	Medians (1st and 3rd quartiles)		p value^b^
		CG	EG	
				
ICDAS II^a^	T0	0.63 (0.24 - 1.26)	0.41 (0.14 - 0.94)	0.0556
	T1	0.30 (0.16 - 0.69)	0.58 (0.34 - 0.85)	
				
Sound teeth	T0	14 (9 - 18.5) *	20.5 (15.3 - 23) ⁑	0.0079
	T1	17 (10.5 - 22) * ⁑	17 (14 - 22) ⁑	
				
Teeth with initial dental caries lesion	T0	3 (0.5 - 8.5) *	2 (0 - 5.8) *	0.0013
	T1	3 (1.5 - 4) *	7 (3 - 9.8) ⁑	
				
Teeth with gingival bleeding	T0	5 (1.5 - 9) *	2 (0 - 6.5) ⁑	0.0096
	T1	6 (2.5 - 11.5) *	5 (3 - 10) *	
				
Gingival bleeding (%)	T0	25 (12.5 - 43.6) *	9.4 (0 - 33) ⁑	0.0029
	T1	27.3 (13.1 - 55) *	20,3 (10.8 - 46.1) *	
				

^a^International Caries Detection and Assessment System; ^b^Kruskal-Wallis test; *no significant difference (p>0.05); ⁑significant difference (p<0.05).

Table 2 shows that there were no statistically significant differences between the groups in T0 (Chi-square, p=0.2557) and T1 (Fisher's exact test, p=1.0), regarding IDCL. There were also no statistically significant differences between the evaluations for the CG (Fisher's exact test, p=0.0859). However, there was a significant increase concerning IDCL in the EG (chi-square, p=0.0033) after the surgical procedure. The same occurred about the presence of gingival bleeding (GB). It increased after one year of surgery in EG (Fisher's exact test, p<0.0001). However, there were no differences between groups in T1 (Chi-Square, p=0.3256).

**TABLE 2:** Initial dental caries lesion (IDCL) and gingival bleeding (GB)’s data, in Experimental (EG) and control (CG) groups, in T0 and T1.

Evaluationperiod	Presence of IDCL			Presenceof GB		
	CG (n=43)	EG (n=46)		CG (n=43)	EG (n=46)	
T0	32 (74.4%)	28 (60.9%)	*	35 (81.4%)	33 (71.7%)	*
						
T1	39 (90.7%)	41(89.1%)	*	40 (93%)	46 (100%)	*
						
	*	(p=0.0033)^**^		*	(p<0.0001)^***^	

*Non-significant difference; **Chi-square test; ***Fisher’s exact test.

 Although there was a significant worsening of the oral condition among the EG participants after bariatric surgery, calculations of relative risks and differences between the incidence rates of EG and CG showed that the procedure did not represent a risk factor for IDCL nor for GB (Table 3).

**TABLE 3:** Risk analysis of bariatric surgery on Initial Dental Caries Lesion (IDCL) and Gingival Bleeding (GB).

	RR^a^	CI^b^ 95%	p value	RRR^c^/RRI^d^	IR^e^ (p value)
IDCL	0.86	0.63 - 1.17	0.3439	14% (RRR)	0.8675
GB	1.14	-	0.4008	14% (RRI)	0.6913

^a^Relative Risk; ^b^Confidence Interval; ^c^Relative Risk Reduction; ^d^Relative Risk Increase; ^e^Incidence Rate.

Linear regression analysis was performed to verify the dependence of BMI and weight loss with the variables under study. In the case of BMI, the obtained model was significant (p<0.0001) and the used variables showed an adjusted R2 of 0.31, indicating that these variables explain 31.1% of the weight variation. Table 4 shows the model from the regression, from where it is possible to establish the following relation: weight=58.6 - (9.5xbariatric surgery) + (5.1xhypertension) - (3.6xnumber of brushing) - (0.33xnumber of healthy teeth). By the equation, it was observed that weight decreases with bariatric surgery, with the greatest number of toothbrushes and healthy teeth, but it increases in patients with hypertension. The other variables were not significant (p>0.05) for the model.

**TABLE 4:** Linear regression analysis considering the Body Mass Index (BMI) regarding the variables that were significant for the model

	Beta	Standard Error	Standardized beta	t	p
Constant	58.6	3.0	-	19.53	<0.0001
Bariatric surgery	-9.5	1.3	-0.45	-7.08	<0.0001
Hypertension	5.1	1.4	0.24	3.70	0.0003
Number of daily dental brushing	-3.6	0.99	-0.23	-3.64	0.0004
Number of sound teeth	-0.33	0.14	-0.15	-2.33	0.0207

**TABLE 5:** Linear regression model considering weight loss regarding significant variables

	Beta	Standard Error	Standardized Beta	t	p
Constant	4.34	1.8	-	2.4	0.017
Bariatricsurgery	42.1	2.2	0.872	19.3	<0.0001
Gender	10.7	3.2	0.151	3.4	0.001
Diabetes	-5.5	2.5	-0.102	-2.3	0.027

For the calculation of the variable 'weight loss', the difference between weight at T0 and T1 was considered, independently of the groups under study. Table 5 shows the regression model, using weight loss as the dependent variable. From the regression model presented, the decrease in participant weight occurs due to bariatric surgery and sex, while the presence of diabetes is related to lower weight loss. Thus, bariatric surgery is undoubtedly the major contributor to weight loss and in men there is a greater loss, but this loss decreases in the presence of diabetes.

## DISCUSSION

The outcomes of this research highlighted that anxiety does not improve 12 months after surgery. Furthermore, there were increases in the number of initial dental caries lesion (IDCL) and sites of gingival bleeding (GB).

 In this study, the anxiety results were not altered by bariatric surgery, differing from some published studies, which found a significant improvement in the levels of this disorder after bariatric surgery^4^ ^,^ ^24^. However, a study that evaluated anxiety through interviews conducted by psychologists, in the preoperative and postoperative periods of 6-12 and 24-36 months, found no significant difference between the evaluations^6^.

Frustrations related to the expectation of expressive weight loss after surgery could explain the persistence of the same levels of anxiety in the postoperative period. In the present study, despite significant weight loss in the postoperative period, 60.9% still remained obese after 12 months of surgery. On the other hand, too much weight loss during this period could also be observed in some patients and, according to de Zwaan et al.^7^, such loss usually results in excess skin and sagging, which may cause physical discomfort, as well as negatively affect the quality of life, self-esteem, body image and physical functioning, making these individuals more anxious.

The analysis of IDCL and GB results showed that, in T0, obese patients who did not undergo bariatric surgery, presented greater impairment than obese patients who had their surgeries already programmed. This may be due to the fact that the participants of the EG were, at the time of the evaluation, under dental care, since the maintenance and/or restoration of oral health, in order to favor satisfactory chewing, a mandatory requirement for the accomplishment of the bariatric surgery in the origin service of these patients. However, while the CG maintained the same condition in T1, the EGpresented worsening in these outcomes after 12 months of surgery. In EG, in T1, it was possible to identify a significant increase in the number of teeth with IDCL, as well as in the number of participants with these lesions. Among the factors that could explain these results, there are certain changes in habits after surgery, especially the feeding ones, regarding the frequency of ingestion and the type of food consumed. In this case, Machado et al.^18^ observed that patients undergoing bariatric surgery developed new eating patterns to contain emotional difficulties, using food of easy ingestion, such as candies, toast, biscuits and other snacks.

Regarding GB, significant increases were found 12 months after surgery in number and in percentage of bleeding sites, as well as in the presence of this variable. These results are in line with the research findings by Sales-Peres et al.^21^, which showed a significant increase in GB after six and 12 months of bariatric surgery. This may be related to changes in both eating and anatomical behavior inherent to bariatric surgery. Like dental caries, behavioral habits, such as increasing the frequency of food intake and the consumption of candies and carbohydrates, which adhere easily to the dental surface, associated with unsatisfactory oral hygiene, may cause deleterious effects on the gingival tissues, since these practices favor the accumulation of bacterial plaque, especially in the tooth-gingival interface.

The influence of variables studied on the participants weight was evaluated through a regression analysis, which showed that lower weight was attributed to bariatric surgery, better oral hygiene and a higher number of healthy teeth, while the presence of hypertension increased according to weight. The dietary pattern of an individual is conditioned, among other factors, to efficient masticatory function, which depends directly on good oral health conditions, being compromised in the presence of diseases such as dental caries and periodontal diseases. In the presence of reduction in masticatory efficiency, it is common to prefer industrialized foods rather than in natural foods, because of its consistency, and therefore easier to ingest. Therefore, a higher number of healthy teeth suggest a greater masticatory capacity, thus allowing the consumption of adequate food. This mechanism could explain the results found in this study, considering that a greater number of healthy teeth are associated to a greater number of daily dental brushings, which constitute the significant oral variables in the model.

Relationship between weight and the hypertension was also observed in this study, but in a positive way, since the weight increased in subjects who presented hypertension. The literature points to a considerably higher prevalence of obesity among hypertensive individuals when compared to normotensive individuals^9^. Mechanism that could explain these findings refers to the greater activation of the sympathetic nervous system, common in obesity, which plays an important role in the pathogenesis of hypertension. High levels of circulating leptin and free fatty acids, low serum levels of ghrelin and adiponectin, and hyperinsulinemia are the main mechanisms involved in the genesis of sympathetic hyperactivity in obesity. Increased sympathetic activity may raise blood pressure via peripheral vasoconstriction and increased renal tubular reabsorption of sodium^16^.

Finally, better results regarding weight loss were attributed to bariatric surgery itself and to males, while diabetics lost less weight after surgery. Although in this study the frequency of male participants was low, which could have compromised their representativeness, other studies presented similar results^2^ ^,^ ^5^ ^,^ ^17^.

The BMI, measured before surgery, has been shown to be a strong determinant of weight loss^5^. Higher initial weight or BMI have been associated with worse weight loss results after bariatric surgery^20^. The initial weight data used in this study refer to the time when the participants entered the bariatric surgery service. However, it is known that many patients need to lose some weight to undergo surgery in order to improve surgical conditions^2^and thus make the procedure safer. Perhaps, in this study, male participants, until the time of surgery, have eliminated more weight than women.

Another explanation for this result refers to the differences in the labor pattern between men and women, who could also have influenced these findings, since, commonly, men tend to perform heavier jobs, which require more energy expenditure, favoring weight loss. Another factor is related to the presence of eating disorders, which was not investigated in this study, however, according to the literature, such disorders, especially compulsive disorders, negatively interfere with weight loss^1^. In addition, according to Espindola & Blay^8^, eating disorders are much less frequent in men when compared to women, which represent 90 to 95% of cases.

The presence of diabetes in the preoperative period had a negative influence on weight loss after bariatric surgery. Converging results were found in other studies^3^ ^,^ ^5^ ^,^ ^17^ ^,^ ^20^. Probable explanation for the negative influence of diabetes on weight loss appears not to be associated with the presence of the disease itself, but with its treatment. With the exception of metformin, medications used to treat diabetes, such as exogenous insulins and sulfonylureas, usually increase circulating levels of insulin. Insulin, in turn, is an anabolic hormone that promotes lipogenesis, stimulation of triglyceride synthesis, differentiation of adipocytes and muscle synthesis. Thus, it is assumed that elevated circulating levels of this hormone may reduce the degree of weight loss after bariatric surgery. There are other factors related to these treatments, such as the increased risk of hypoglycemia, which in turn stimulates the increase of caloric intake in order to prevent and/or treat these episodes. Also, the retention of sodium and water, which is a direct effect of the insulin in the distal tubule of the kidney, may also occur as a reflex of these treatments^3^ ^,^ ^5^.

The results found in this study confirmed the hypothesis that bariatric surgery interferes negatively in IDCL and GB, of bariatric patients after 12 months of surgery, but contradicts that the same procedure is a risk factor for the outcomes studied and that promotes improvement in anxiety scores. However, some limitations should be considered. The relatively small sample size and lack of matching of the groups may have compromised the results found and, in addition, there was a reasonable loss of participants during the study. To assess anxiety, a self-administered questionnaire was used, which does not allow an evaluation of the subjective and behavioral aspects of the individuals^22^. Also, some investigated variables, especially behavioral ones, depended on the patients' reports, such as the number of daily brushings, which may have compromised the reliability of the data. The fact that the sample was composed of a population restricted to a certain geographic location and, for the most part, by women, makes it difficult to generalize the results. Consideration should also be given to the relatively short follow-up time and the non-inclusion of some behavioral variables in the postoperative period.

Despite the limitations pointed out, there were no studies in the literature that simultaneously studied obesity, anxiety, early caries lesions, gingival bleeding and bariatric surgery. Another advantage of this study is its longitudinal design, which allows studying the changes that occur in the population during the period in which it was followed. However, more research is needed using larger samples, more accurate instruments for the diagnosis of anxiety, and longer follow-up times. The inclusion of other variables related to the disorders studied is also fundamental for the elucidation of the supposed relations.

## CONCLUSION

Bariatric surgery does not promoted improvement in anxiety scores after 12 months of the procedure, besides favoring a worsening of the oral condition, in relation to initial lesions of dental caries and gingival bleeding. However, this procedure did not constitute a risk factor for the aforementioned oral outcomes, and it is currently one of the best alternatives for the treatment of severe obesity and its comorbidities, with results of weight loss maintained in long term and consequent decrease of death risk from diseases, especially the cardiovascular ones. Furthermore, it was also observed that higher body weight was associated with less healthy teeth and daily tooth brushing. These results reinforce the need for multiprofessional team follow-up, including dental care, for both obese and bariatric patients, so they may proper benefit surgery for weight reduction and related diseases with quality of life, through the control of the damage that result from the surgical procedure.
